# Impact of a Temperature-Monitored Blood Transfusion Network on Reducing Blood Wastage

**DOI:** 10.7759/cureus.89255

**Published:** 2025-08-02

**Authors:** Fidha Ashraf, Arshad Kalathil Ashik, Ashfaq Ashraf Chathoth, Asad Luqmani

**Affiliations:** 1 Family Medicine, Ahalia Diabetes Hospital, Palakkad, IND; 2 Department of Mechanical Engineering, Imperial College London, London, GBR; 3 Department of Management Studies, Indian Institute of Science, Bengaluru, IND; 4 Department of Haematology, Imperial College Healthcare NHS Trust, London, GBR

**Keywords:** blood bank, blood transfusion, blood wastage, internet of things, temperature monitored

## Abstract

Lack of availability of blood for transfusion is associated with significant mortality rates across the world. Blood collection and storage can be managed either in centralised or decentralised facilities. India has a decentralised blood bank network supply chain, which results in blood wastage due to the lack of data to decide on the quality and suitability of blood for transfusion. In this study, we introduce a temperature-monitored blood bank supply chain that tracks the temperature log of blood from collection to delivery. An algorithm was developed according to the World Health Organization guidelines (*Design Guidelines for Blood Centres*) to predict the usability of blood. This research aims to measure the difference in blood wastage by tracking and analysing the end-to-end temperature data. Testing was carried out over a period of two years in two cycles of six months each to evaluate the effectiveness of the solution. It was found that using this solution reduced blood wastage by 68% at the blood storage centre. Statistical analysis using a paired t-test showed a significant reduction in blood wastage, with a p-value of 0.044 and a large effect size (Cohen’s d = 1.09). Implementing this methodology at a larger scale can help identify temperature breaches across the blood supply chain and ensure safe blood is available for transfusion.

## Introduction

Blood transfusion is an essential component of medical practice, saving millions of lives every year [1,2]. Whether it is to treat chronic anaemia, manage trauma and major haemorrhage, or support patients undergoing major surgeries, blood transfusions provide essential medical support [3,4]. It is estimated globally that 119 out of 195 countries lack an adequate blood supply to meet their needs [5]. Most of the countries that do not have enough blood availability fall under low-income countries. India, despite being one of the largest nations with a growing healthcare sector, faces significant challenges in meeting blood demand [6]. Even though India has a blood need-to-supply ratio of approximately 1:4, in absolute terms, India has the largest unmet blood need [5]. This large-scale shortage of blood in India has pushed several physicians to request the allowance of the banned unbanked direct blood transfusion (UBDT) to address the non-availability of blood units in rural India [6,7]. However, allowing UBDT can expose patients to life-threatening infections such as human immunodeficiency virus, hepatitis B, hepatitis C, syphilis, and other transfusion-transmitted diseases (TTDs) [8].

Currently, in India, there are three types of blood storage facilities: hospital blood bank (HBB), independent blood bank (IBB), and blood storage centre (BSC). HBB and IBB act as the first point of contact in the blood transfusion supply chain; only these two blood banks have the permission to collect and store blood from donors [9]. BSCs are secondary storage centres set up near hospitals that do not have a blood bank unit (blood banks have been officially renamed by the Government of India as ‘blood centres’; however, in this article, we use the old terminology for clarity and to distinguish them from BSCs mentioned in this work). Usually, BSCs are located within a distance of one hour of travel time to the hospital and may serve more than one hospital [9,10]. Each of the BSCs is connected to a mother blood bank (MBB) that supplies the needs of the attached BSC, and there can be multiple BSCs attached to a single MBB [10,11]. An MBB can be either an HBB or an IBB that supplies blood to the attached BSC.

A robust and efficient blood bank supply chain is essential to provide quality blood in time for patients’ needs [12,13]. Currently, most regions in India have a broken blood supply chain, all the data is recorded manually, and there is no way to ensure that the blood is collected and stored within the World Health Organization (WHO)-approved temperature limits of 2-6 °C [14-18]. The absence of this data can result in blood wastage and affect the quality of the delivered blood.

MBBs are large blood bank facilities where there is minimal to no wastage due to the high demand for blood. However, BSCs that are attached to an MBB for supplying the needs of remote hospitals have considerable blood wastage [15]. According to WHO guidelines, blood can be stored safely for 42 days in the temperature range of 2-6 °C [16]. In India, BSCs store blood proportionate to the size of the hospitals they serve. The blood bank guidelines specify that if a blood bag is not utilised within 30 days in a BSC, it should be sent back to its respective MBB [17]. This guideline was introduced to reduce blood wastage at BSCs, and if followed properly, the returned blood bags can be utilised for transfusion at an MBB where there is a high demand for blood. However, mother blood banks refuse to accept blood back from BSCs due to the absence of storage condition data. Without the temperature data logging during transport and storage conditions, it is impossible for the MBB to ascertain the quality of the blood. Therefore, as a safety measure, blood is stored until its expiry in a BSC and is discarded if the particular blood unit is not utilised. This process has led to an increase in wastage at BSCs [19,20].

In India, a significant amount of blood is wasted during transportation between blood banks and hospitals due to the absence of reliable temperature data to assess its usability. Currently, no standard solutions exist to determine the quality of blood after transport, leading blood banks to reject blood units that have been returned from hospitals. This study introduces a temperature-monitored blood supply chain network designed to reduce such wastage by continuously logging the temperature of blood from initial storage to final delivery. The objective is to evaluate the impact of temperature monitoring on reducing blood wastage at blood banks. A pre- and post-intervention analysis was conducted, and an algorithm was developed to predict the usability of transported blood. This solution has the potential to ensure the quality of delivered blood, minimise clerical errors, and enhance the operational efficiency of blood banks in stock management.

## Materials and methods

Methodology

The proposed methodology involves an Internet of Things (IoT)-enabled centralised blood bank information system designed to track and monitor the temperature and location of each blood bag throughout the supply chain. To ensure efficient testing, every bag was assigned a unique identifier. This was achieved by attaching a radio-frequency identification (RFID) tag containing a unique number to each bag. RFID scanners and temperature sensors were then installed in blood bank refrigerators and transport containers. The scanners tracked the location of each bag, while the sensors recorded real-time temperature data.

When a new blood bag arrives at the MBB, it is first transferred to a storage unit. The RFID scanner attached to the unit automatically records the bag's entry and transmits this data via the internet to the centralised monitoring system. Simultaneously, the system initiates real-time temperature monitoring using the storage unit's embedded sensor.

If the blood bag is dispatched to a BSC, the system immediately detects its removal from the MBB storage. It then identifies the assigned transport container and begins recording the updated temperature data during transit. Upon arrival at the BSC, the system recognises the new storage container and starts logging the corresponding environmental data. This same process repeats if the bag is returned to the MBB. As this study did not involve any direct patient participation, ethical considerations were waived. However, all the devices used in this study were calibrated by the National Accreditation Board for Testing and Calibration Laboratories (NABL) standards for testing in medical settings in India, under reference number TM/190628B/07.

This approach ensures continuous monitoring of each blood bag’s temperature, from the time of collection at the blood bank to its storage, transportation, and eventual use in remote locations or its return to the blood bank, if unused.

Testing environment

The work was carried out between the MBB, General Hospital Thiruvananthapuram (GHT), and an attached BSC, Taluk Headquarters Hospital, Parassala (THHP). GHT is one of the main blood banks in the Thiruvananthapuram district and supplies blood to six BSCs, with THHP being one among them. GHT is one of the largest hospitals in South Kerala, with 769 beds, and has an annual blood red cell usage of 2305 units. THHP with 119 beds is situated 35 km away from GHT and utilises 216 units of red cell units annually.

To test the solution, the transport container, along with the refrigeration units at both hospitals, was fitted with the monitoring device. GHT had the monitoring software installed to track the location and quality of blood. With our solution, a technician at GHT was able to monitor the blood data at the storage centre, receive alerts in case of a temperature breach, and request blood be returned to the MBB if not used within 30 days of collection.

Only red blood cells (RBCs) that have a shelf life of 42 days were considered for this study. All references to blood bags in this work refer to RBC units. The overall testing was completed over a period of two years, from August 2019 to July 2021, in two different cycles. The first test cycle occurred from August 2019 to January 2020, and the second cycle from August 2020 to January 2021. In the first cycle, the data related to the availability and discarding (wastage) of blood was collected using the pre-existing methods before the implementation of our solution, and in the second cycle, the same data was recorded with our blood-monitoring solution in place. The time gap was introduced to get the same month-to-month comparison.

Algorithm used to classify blood suitability for transfusion

**Figure 1:**
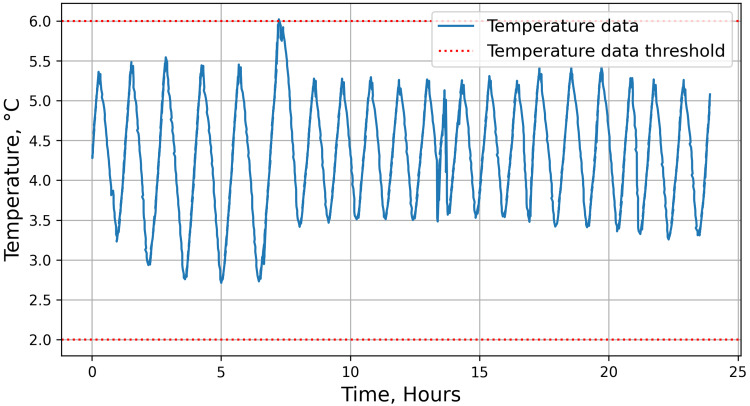
Sample temperature data recorded by the device for a time period of 24 hours. The monitored temperature data is further used to determine the quality of blood using WHO guidelines.

The IoT-enabled monitoring system tracks the temperature of blood, starting from the blood collection at a blood bank. We derived local guidelines for safe blood from the existing WHO guidelines [18]. To begin with, the blood bags that are stored at 2-6 °C but not utilised within 42 days are classified as unsafe for transfusion. Blood stored at temperatures exceeding or subceeding the above-mentioned safe limit for longer than 30 minutes is also categorised as unsuitable for transfusion [21]. Finally, the absence of a temperature data reading for more than 30 minutes for a given blood bag at any point during the transfusion cycle leads to it being labelled unsuitable. Figure 1 shows the temperature reading of a blood bag during its storage, recorded over a period of 24 hours.

Statistical analysis

To evaluate the effectiveness of the implemented solution in reducing blood wastage, a statistical analysis was performed using paired data from two corresponding six-month periods (August to January), one year apart.

A paired t-test was employed to determine whether there was a statistically significant difference in the number of discarded bags before and after the intervention [22]. This test was chosen because it compares means from the same units (months) measured pre- and post-implementation. The differences in the number of discarded units were computed for each matched month, and the resulting mean difference was tested for statistical significance. A p-value less than 0.05 was considered statistically significant.

To quantify the magnitude of the observed change, Cohen’s d was calculated for paired samples [23]. The difference between the mean of the two groups was divided by the pooled standard deviation of the data. This standardised effect size allowed for an assessment of how meaningful the reduction in discarded units was, beyond mere statistical significance. The calculated Cohen’s d was interpreted using conventional thresholds (0.2 = small, 0.5 = medium, 0.8 = large, 1.0+ = very large effect).

All statistical calculations were performed using Python (NumPy, SciPy, Pandas, and Seaborn libraries), and data were visualised using box plots with overlayed mean ± standard error of the mean (SEM).

## Results

Comparison of blood wastage

To understand the amount and reasons for blood wasted at the test facility, the blood wastage data from January to December 2019 was studied. It was found that 60 bags were discarded from the BSC THHP during this period, as the storage date had expired. A more detailed investigation into wastage data at the BSC revealed that no bags were returned to the MBB due to insufficient data on the storage conditions of the blood.

**Figure 2:**
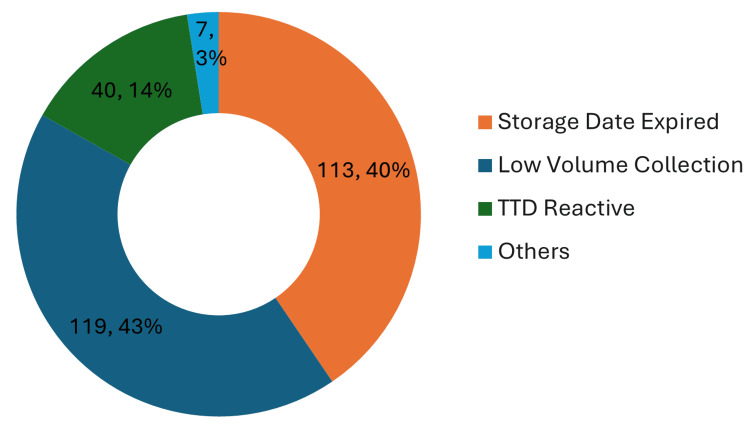
Comparison of blood wastage at MBB GHT in year 2019, classified into different categories TTD: transfusion-transmitted disease; MBB: mother blood bank; GHT: General Hospital Thiruvananthapuram

To derive a holistic understanding of the blood wasted along the entire blood bank supply chain, the discard data at MBB was analysed and classified into four categories: storage date expired, low volume collection, TTD reactive, and others. Blood bags that were discarded because they could not be utilised within 42 days of their collection were classified as ‘storage date expired’. Cases where a donor was unable to donate sufficient blood for storing and transfusion were categorised under ‘low volume collection’. Blood bags are also discarded when they test positive for any TTD. All discarded units where sufficient data was not available were classified under ‘others’. Figure 2 shows the breakdown of blood wastage; most of the blood bags were discarded as the collection volume threshold could not be met; 14% of the discarded units were due to testing positive for a TTD, and for the remaining 3%, the discard data made no mention of the reason. The relevant data set for this study lies in the units discarded due to exceeding the expiry date, which amounted to 41%.

Reduction in blood wastage following implementation of the solution

The temperature-monitored blood transfusion supply chain solution between an MBB and BSC was tested over a period of two years, in two cycles of six months each. To measure the effectiveness of our solution, the number of blood bags discarded was selected as the parameter to measure the impact of the intervention. The first cycle measured the historical blood wastage data prior to our intervention, and in the second cycle, wastage data was recorded after implementing the solution.

**Figure 3:**
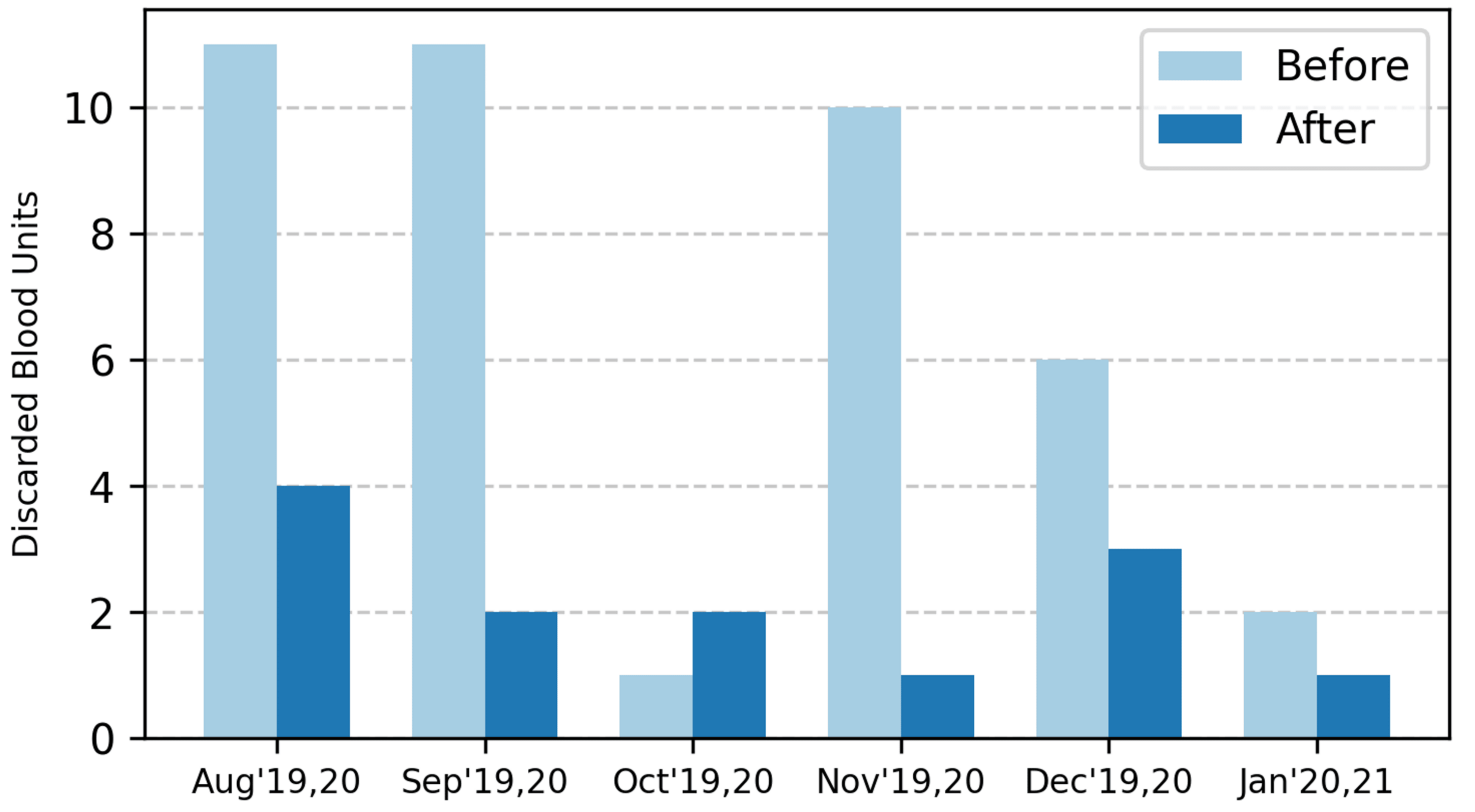
Comparison of blood bags discarded over a period of six months from August to January in 2019 and 2020, respectively The first test cycle occurred from August 2019 to January 2020, and the second from August 2020 to January 2021. In the graph, 19 signifies 2019, 20 signifies 2020, and 21 signifies 2021.

BSC THHP receives all of its blood stock from MBB GHT. A total of 389 blood bags were examined for the study, with 210 blood bags before the intervention and 179 bags after the intervention. Figure 3 shows the number of blood bag units discarded before and after the implementation of the technology. It was found that between August 2019 and January 2020, a total of 41 blood bags were discarded, with the highest number of 11 bags in each of August and September 2019. The blood bags discarded in the six-month period after the installation of our solution reduced blood wastage by 68%, to just 13 bags. The maximum number of bags discarded in any one month after implementing the solution was four bags in August 2020.

Statistical outcomes

Descriptive statistics for the number of discarded blood bags before and after the implementation of the solution are presented in Table 1. The mean number of discarded units decreased from 6.83 (±1.85 SEM) before implementation to 2.17 (±0.48 SEM) after implementation. This reduction was accompanied by a notable decrease in variability, as reflected by the standard deviation, which dropped from 4.31 to 1.17 units.

**Table 1:** Statistical measures before and after implementation SEM: standard error of the mean

Statistical measure	Before implementation	After implementation
Mean	6.83	2.17
Standard deviation	4.54	1.17
SEM	1.85	0.48

**Figure 4:**
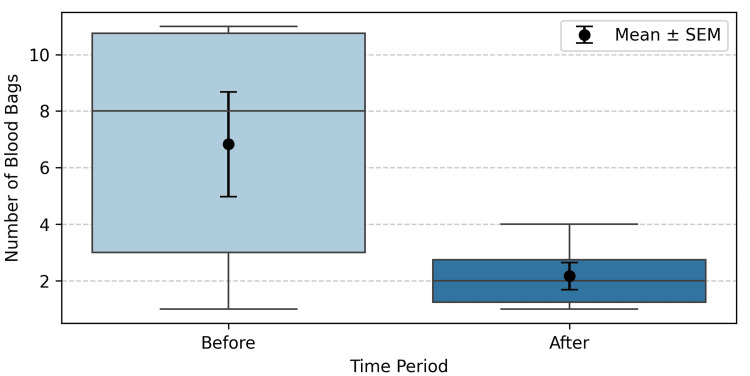
Box plot showing the mean ± SEM of discarded blood bag units before and after the implementation of the solution SEM: standard error of the mean

Figure 4 displays a box plot comparing the number of discarded blood bag units before and after the implementation of the solution. It can be observed that after implementation, the data showed both a lower median and reduced variability compared to the period before implementation.

A two-tailed paired t-test was conducted to assess whether there was a significant difference in the mean number of discarded blood units before and after the intervention. The null hypothesis stated that there was no difference in the mean number of discarded units. The test yielded a p-value of 0.044 (t(5) = 2.67), indicating statistical significance at the 5% level. Thus, the null hypothesis of no difference was rejected, supporting the conclusion that the intervention led to a meaningful reduction in discarded blood products. These results suggest that the implemented solution significantly improved the efficiency of blood usage and reduced wastage.

To quantify the magnitude of change in blood wastage, Cohen’s d was calculated for paired samples. The resulting Cohen’s d was 1.09, indicating 'a very large' effect size according to standard benchmarks, and highlighting that the solution was not only statistically significant but also clinically relevant.

## Discussion

In India, high blood wastage at BSCs occurs because MBBs often fail to retrieve units before expiry due to poor connectivity and lack of temperature data [24,25]. However, the lack of data on temperature during transportation makes it difficult to assess blood quality, leading MBBs to reject any units that have been sent out for transport. In this study, we addressed this issue by attaching temperature sensors to all blood storage units, including the transport containers, and assigning a unique RFID card to each blood unit. Afterwards, using the proposed algorithm, the quality of the blood was predicted.

With the temperature-monitored supply chain network for blood transport, we demonstrated a significant impact in reducing blood wastage at the BSC. Over a period of six months, we were able to reduce wastage by 68%. Statistical analysis showed a high impact after implementing the solution, with a p-value of 0.044 and a Cohen’s d value of 1.09. The reduction in wastage is attributed to the availability of data for decision making, which was not available previously. Identifying the temperature limits through which the blood travelled, combined with the WHO guidelines for safe blood transport, helped in determining the quality of the blood.

On analysing the data on the total blood units discarded (Figure 2), it was observed that 43% of the blood was wasted due to low collection volume. Directly reducing this wastage is not possible under the scope of our study intervention. However, the current methodology can be expanded to record all the information of donors and their previous blood donation histories, and with this data, the healthcare provider will be able to see if a donor has any previous history of low volume collection. Utilising the blood donor history information, the blood bank can make an informed decision whether to collect from such a donor.

According to current practices in many rural areas of India, if a blood unit tests positive for TTDs, it is discarded; however, the donor is often not informed of their condition. This gap in communication is frequently due to the additional burden placed on healthcare personnel. Our analysis indicates that 14% of blood discards are due to positive TTD results, and previous studies have highlighted the significant impact of TTDs on blood transfusion services [26,27]. While it may not be possible to eliminate such discards, informing the affected donors can help reduce the financial burden on the healthcare system, limit disease transmission, and enable early diagnosis and treatment. The proposed solution can be expanded to store donor information and integrated with the electronic medical record, allowing health authorities to notify donors if their blood tests positive for any infectious disease.

Currently, the proposed solution to reduce blood wastage in rural areas has been tested in a setting where the distance between the MBB and BSC is 35 km. However, testing this methodology in a resource-limited setting where the facilities are much farther apart will provide insights into the impact on a larger scale. Also, due to resource constraints, the solution was tested over a one-year period (six months before and six months after installation). Extending the testing period would help better understand its effect on blood bank operations and wastage reduction.

## Conclusions

Blood wastage in rural India remains high, primarily due to the lack of reliable information to assess blood quality. In this study, we addressed this issue by testing an end-to-end temperature-monitored blood supply chain network. This solution targets the long-standing problem of blood being discarded due to unavailable temperature data during transport. The methodology was evaluated over a six-month period between an MBB and a BSC, resulting in a 68% reduction in blood wastage. Statistical analysis confirmed the effectiveness of the solution with a significant impact. Implementing this methodology at a larger scale will help to easily identify temperature breaches across the blood supply chain and ensure the availability of safe blood for transfusion. Apart from reducing blood wastage, digitalising the supply chain will reduce the burden on the hospital staff to maintain multiple registers and minimise clerical errors.
